# Viral Toxin NS1 Implication in Dengue Pathogenesis Making It a Pivotal Target in Development of Efficient Vaccine

**DOI:** 10.3390/vaccines9090946

**Published:** 2021-08-25

**Authors:** Grégorie Lebeau, Alisé Lagrave, Eva Ogire, Lauriane Grondin, Soundary Seriacaroupin, Cédric Moutoussamy, Patrick Mavingui, Jean-Jacques Hoarau, Marjolaine Roche, Pascale Krejbich-Trotot, Philippe Desprès, Wildriss Viranaicken

**Affiliations:** Unité Mixte Processus Infectieux en Milieu Insulaire Tropical, Plateforme Technologique CYROI, Université de la Réunion, INSERM U1187, CNRS UMR 9192, IRD UMR 249, 94791 Sainte Clotilde, La Réunion, France; gregorie.lebeau@univ-reunion.fr (G.L.); alise.lagrave@hotmail.fr (A.L.); eva.ogire@univ-reunion.fr (E.O.); laurianegrondin19@gmail.com (L.G.); 37000589@co.univ-reunion.fr (S.S.); 36004347@co.univ-reunion.fr (C.M.); patrick.mavingui@cnrs.fr (P.M.); jean-jacques.hoarau@univ-reunion.fr (J.-J.H.); marjolaine.roche@univ-reunion.fr (M.R.); pascale.krejbich@univ-reunion.fr (P.K.-T.)

**Keywords:** arbovirus, dengue, viral hemorrhagic fever, viral immunopathogenesis, viral toxin, NS1, dengue vaccine strategies

## Abstract

The mosquito-borne viral disease dengue is a global public health problem causing a wide spectrum of clinical manifestations ranging from mild dengue fever to severe dengue with plasma leakage and bleeding which are often fatal. To date, there are no specific medications to treat dengue and prevent the risk of hemorrhage. Dengue is caused by one of four genetically related but antigenically distinct serotypes DENV-1–DENV-4. The growing burden of the four DENV serotypes has intensified both basic and applied research to better understand dengue physiopathology. Research has shown that the secreted soluble hexameric form of DENV nonstructural protein-1 (sNS1) plays a significant role in the pathogenesis of severe dengue. Here, we provide an overview of the current knowledge about the role of sNS1 in the immunopathogenesis of dengue disease. We discuss the potential use of sNS1 in future vaccine development and its potential to improve dengue vaccine efficiency, particularly against severe dengue illness.

## 1. Dengue Disease

Dengue disease has become one of the most significant mosquito-borne viral diseases worldwide. The global incidence of dengue has dramatically increased over the last decades causing a major public health problem in tropical and subtropical regions where endemicity relates to the profusion of *Aedes aegypti* and, to a lesser extent, of *Aedes albopictus* as major vectors for dengue virus (DENV) transmission. The four serotypes of DENV, DENV-1–DENV-4, sharing 60–80% homology in their genomic sequences, can cause flu-like illness, but some individuals can experience severe plasma leakage associated with exacerbated inflammatory responses leading to potentially fatal shock. The mechanisms of severe dengue are poorly understood and presumably multifactorial with viral and host factors having significant roles. The immune status of patients might play a key role in the risk of severe dengue. Indeed, the antibody-dependent enhancement (ADE) or the original antigenic sin phenomenon have been associated with the development of severe dengue which relates to secondary infection with a DENV serotype different of that responsible for the primary infection. Thus, preexisting immunity against DENV could be associated with the development of severe forms of dengue disease during a secondary infection. To date, no specific treatments nor therapies are available for clinical management of severe dengue disease.

**Figure 1:**
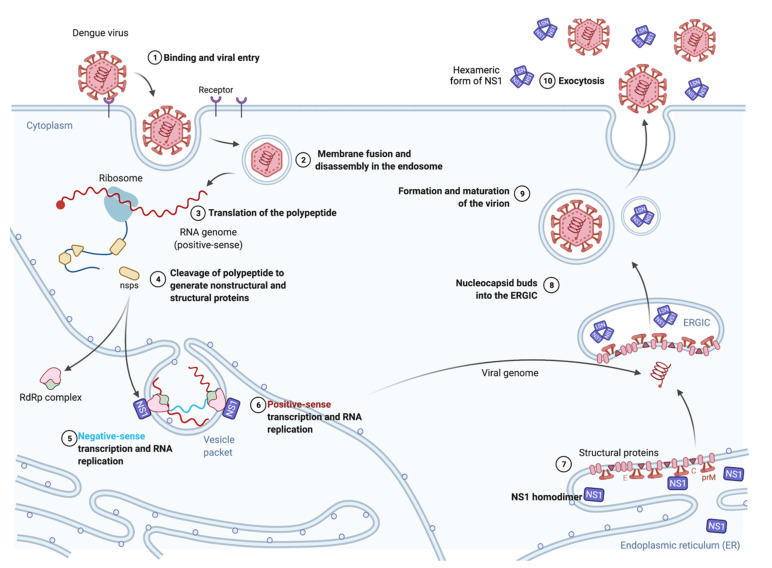
DENV NS1 protein involvement in the viral cycle. Nonstructural protein 1 (NS1) of flaviviruses has been described with diverse functions during the viral lifecycle (1–10). After its cleavage from the polyprotein (3,4), NS1 forms homodimers associated with viral RNA complexes (5,6) and contributes to viral morphogenesis through interactions with the prM and E proteins (7,8). Furthermore, thanks to its hydrophobic properties and membrane affinity, NS1 participates in the formation of vesicle packets, which are essential structures hosting viral replication machinery. Interestingly, among the nonstructural proteins, NS1 has a particular fate. Indeed, the soluble hexameric form of NS1 circulates in infected individuals. The release of soluble NS1 requires protein transport into the ERGIC (ER–Golgi intermediate compartment) (10).

DENV is a positive single-stranded RNA virus which belongs to the *Flavivirus* genus of the Flaviviridae family sharing great similarity with other medically important arboviruses such as yellow fever virus, West Nile virus and Zika virus [1]. DENV has an enveloped viral particle of approximatively 50 nm in diameter; its 11 kb genome is contained within a 30 nm dense core which is surrounded by a lipid bilayer [1,2,3]. The DENV infection lifecycle is initiated by the recognition of virus particles through attachment factors and receptors at the cell surface. Internalized virus particles are trafficked to the endosomal compartment where low pH-mediated fusion between viral and intracellular membranes causes the release of the nucleocapsid into the cytosol [1,3,4,5]. Once released from the nucleocapsid, the free genomic RNA is translated into a long polyprotein which is co- and post-translationally processed to produce the three structural proteins C, prM/M and E followed by seven nonstructural proteins NS1, NS2A/B, NS3, NS4A/B and NS5. New DENV particles are produced within the endoplasmic reticulum (ER, Figure 1). At the early stages of DENV replication, the nonstructural proteins are involved in both viral RNA replication and the subversion of antiviral innate immune responses in the host cell [6]. At the later stages, viral assembly occurs at the ER–Golgi intermediate compartment (ERGIC). The assembled virus particles are trafficked through the secretory pathway and then released as infectious virions by exocytosis.

The NS1 glycoprotein is the only nonstructural viral protein detected in the bloodstream of dengue patients during the acute phase of the infection. Several reports highlight the involvement of soluble NS1 (sNS1) in the pathogenesis of severe dengue [7,8,9,10,11,12]. In this review of the role of NS1 in the immunopathogenesis of dengue, particular attention is paid to the reasons for the current consideration of sNS1 in the design of dengue vaccine candidates.

## 2. Biology and Function of the DENV NS1 Protein

The DENV NS1 protein (352 amino acids) is divided into three functional regions designated as the β-roll, wing and β-ladder domains [13]. After synthesis and proteolysis of the viral polyprotein, NS1 is glycosylated on residues N130 and N207. The post-translational maturation process results in the formation of homodimeric NS1 which, together with other NS proteins, forms viral replication complexes responsible for copying the viral genome (Figure 1). As a result of its hydrophobic properties and membrane affinity [13], NS1 acts as a scaffolding protein, leading to the formation of vesicle packets, a structure known to host viral replicase machinery [14,15]. During the DENV infection, dimeric NS1 has the ability to interact with other viral proteins such as prM and E [14] as well as NS4A [16,17]. Such interactions are involved in viral RNA replication [17] and virus assembly [14].

A proportion of mature NS1 glycoproteins are transported through the Golgi compartment to reach either the plasma membrane as a membrane-associated homodimer or the extracellular compartment as hexameric lipoprotein particles (Figure 1). Secreted soluble NS1 (sNS1) exhibits great structural similarity with high-density lipoproteins carrying triglycerides, cholesteryl esters and phospholipids [18]. During the acute phase of the DENV infection, high levels of sNS1 circulate in the bloodstream, up to 50 µg·mL^−1^, leading to the development of diagnostic kits based on the immunocapture of sNS1 [19]. A relative positive correlation between antigenemia to sNS1 and severity of the DENV infection has been documented [20].

## 3. Secreted Soluble NS1 Contributes to the Pathogenesis of Severe Dengue

During the last decade, a great effort has been made to better understand the role of sNS1 in the pathogenesis of severe dengue. It is now admitted that sNS1 contributes to vascular leakage that is indicative of dengue severity. Indeed, sNS1 has the ability to trigger hyperpermeability of human endothelial cells. After secretion, freely circulating sNS1 is able to bind endothelial cell surfaces, leading to overexpression of sialidases (Neu 1, Neu 2, Neu 3) and heparanase by endothelial cells. This results in disruption of the endothelial glycocalyx layer and therefore contributes towards barrier dysfunction and plasma leakage [12,21]. This mechanism has been confirmed in several endothelial cell lines where a decrease in the *trans*-endothelial electrical resistance is observed in response to NS1 from all four serotypes of DENV [12]. In addition to the disruption of the endothelial glycocalyx layer, rearrangement of intercellular junctions has to be discussed. Protein sNS1 disrupts tight and adherens junctions in a cytokine-independent manner. Several intercellular junction proteins are concerned, including VE-cadherin or zonula occludens-1, possibly through clathrin-mediated internalization and/or phosphorylation [15].

**Figure 2:**
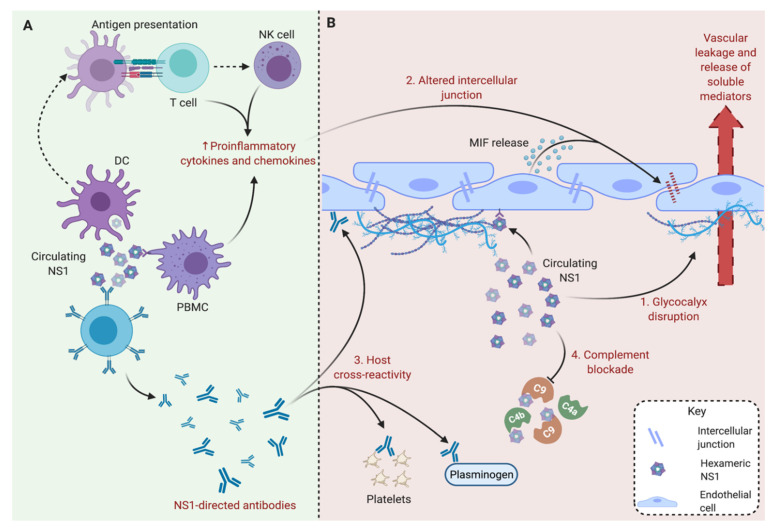
Secreted NS1 implications during the DENV infection. (**A**) NS1-directed immune response. During the infection, the hexameric form of NS1 is released by infected cells and triggers humoral and cell-mediated immune response. B cells produce NS1-directed antibodies which participate in NS1 clearance from blood. However, these antibodies are also involved in pathological processes. On the other hand, peripheral blood mononuclear cells (PBMC) like T cells and NK cells produce proinflammatory cytokines (including interleukin 6 and tumor necrosis factor) and chemokines that participate in inflammation mounting. (**B**) NS1-mediated pathogenesis. Circulating NS1 is directly linked to glycocalyx disruption (1) as well as macrophage migration inhibitory factor (MIF) release by endothelial cells through toll-like receptor 4 (TLR4) signaling. MIF, as other proinflammatory and vasoactive mediators released by immune cells in response to NS1, leads to altered intercellular junctions between endothelial cells (2). In the course of severe dengue, NS1 has the ability to contribute to vascular leakage and cytokine storm. Furthermore, soluble NS1 is associated with production of cross-reactive antibodies that target host endothelial cells, platelets and plasminogen (3). Finally, NS1 may participate in immune evasion through blockade of complement factors such as C4b and C9.

Besides cytokine-independent mechanisms, DENV sNS1 also has the ability to induce expression of vasoactive cytokines, notably IL-6 and TNF-α, which may be associated with the development of vascular disorders in severe dengue (Figure 2B) [9]. Expression of cytokine macrophage migration inhibitory factor (MIF) as part of the response of endothelial cells to sNS1 might play a key role in intercellular junction impairment and subsequent vascular leakage (Figure 2B). Indeed, MIF participates in cell-to-cell contact disruption through internalization and autophagic degradation of intercellular junction proteins, including zonula occludens-1 and vascular endothelial cadherin factors [22,23]. Lastly, sNS1 might play a role in viral immune evasion strategies, mostly through complement blockade (Figure 2B). Indeed, DENV sNS1 functions as a complement-fixing protein for several complement pathway components (C4, C4b, C9 and mannose-binding lectin) [7,8,24,25] and regulators (vitronectin) [25]. Consequently, these interactions have an impact on complement activity and complement-mediated neutralization [15]. In conclusion, the fact that sNS1 has been identified as a viral factor of virulence comparable to a toxin makes it a target of great interest for dengue vaccine strategy.

## 4. Soluble NS1 as a Significant Focus for Dengue Vaccine Strategies

### 4.1. Immunity to the DENV NS1 Protein

**Figure 3:**
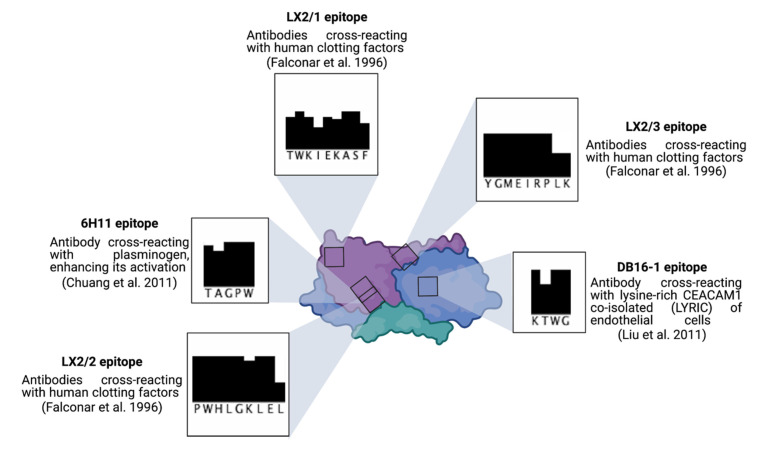
NS1 epitopes in relation to autoantibodies. The NS1 protein is found in circulation during the infection; thus, the existence of molecular mimicry between conserved NS1 epitopes and host proteins may lead to the production of autoantibodies. For instance, the recognition of NS1 epitopes LX2/1, LX2/2 and LX2/3 can engender antibodies cross-reacting with human clotting factors [28], whereas, NS1 epitope KXWG leads to the production of the DB16-1 antibody cross-reacting with lysine-rich CEACAM1 co-isolated (LYRIC) of endothelial cells, promoting cell death by apoptosis [26]. Finally, the 6H11 antibody, which recognizes the NS1 TAGPW epitope, also interacts with plasminogen and enhances its activation [29]. Wing domain (blue), β-roll domain (green), β-ladder domain (purple). Black bars illustrate the conservation degree of the epitope sequence across dengue virus serotypes and genotypes.

At the onset of the DENV infection, sNS1 elicits a potent humoral and cell-mediated immune response (Figure 2A). The NS1-directed antibody response is essentially based on the recognition of B cell epitopes located in the wing and C-terminal regions as the immunodominant domains of hexameric NS1 lipoparticles. The NS1-directed antibodies participate in sNS1 clearance in the bloodstream during the DENV infection and are beneficial to protecting against severe dengue, limiting sNS1-associated dengue immunopathogenesis. However, these antibodies could also be involved in the pathology of severe dengue. As part of dengue immunopathogenesis, sNS1-directed antibodies have the ability to interact with host proteins, presumably as a result of molecular mimicry, as a mechanism of the autoimmune disease associated with the DENV infection (Figure 3) [26,27,28]. Self-antigen recognition by sNS1-directed antibodies might play a role in the triggering of apoptosis and complement-dependent cell cytotoxicity in endothelial cells [26]. Consequently, humoral immunity to DENV sNS1 could be a key effector of coagulopathy observed in dengue patients through activation of plasminogen and platelets which both participate in thrombocytopenia.

On the other hand, the anti-NS1 cell-mediated response is based on DENV sNS1 antigen recognition by peripheral blood mononuclear cells via pattern recognition receptors (PRRs). Activated PRRs from the toll-like receptors family have the ability to trigger the release of major proinflammatory cytokines and chemokines involved in the DENV immunopathogenesis [9,11]. Protein sNS1 has been observed to activate TLR4 and the downstream signaling pathway, leading to IL-6 and IL-8 production, as well as IL-1β and TNF-α expression [11]. Furthermore, TLR6^−/−^ mice exhibited higher survivability in the presence of sNS1 [30]. However, these data should be interpreted with caution because NS1-mediated TLR2/6 activation was observed with recombinant NS1 proteins produced in bacteria but not in invertebrate cells [31].

### 4.2. The Current Challenges for Dengue Vaccine Development

Development of a safe and effective vaccine against all four serotypes of DENV is challenging because of limited understanding of the mechanisms of severe dengue in relation to the immunopathogenesis of the disease. To date, the only licensed dengue vaccine currently available on the market is Dengvaxia developed by Sanofi Pasteur and registered by regulatory authorities in 20 dengue-endemic countries, the European Union and the United States. This is a live-attenuated tetravalent dengue vaccine which contains DENV E and prM genes from the four serotypes inserted in the yellow fever 17D backbone. The objective of this vaccine is to induce preventive humoral and cell-mediated immune responses [32,33]. However, it has recently revealed some major weaknesses. Firstly, it has unequal efficacy against the four DENV serotypes and elicits only limited protection against serotype 2 DENV infection. Secondly, efficacy of Dengvaxia is effective only in the individuals who have been previously infected with DENV. Lastly, the vaccination in children younger than nine years of age was associated with an increased incidence of hospitalization for severe dengue [34]. Although neutralizing E-directed antibodies are assumed to be the main correlate of protection against the DENV infection, the importance of NS proteins for developing an effective dengue vaccine merits greater consideration. Indeed, T cell-based immunity is necessary in controlling the DENV infection, and most of the key targets of these responses are located in the NS proteins [35,36]. Indeed, nonstructural proteins should be part of the vaccine approach, especially sNS1 which exists as circulating hexameric lipoparticles. Given the significant role of sNS1 in the immunopathogenesis of severe dengue, lack of NS1-associated immunity could be a possible explanation for the limited performance of vaccine candidates essentially based on the expression of DENV structural proteins prM and E [12].

### 4.3. DENV Vaccine Candidates Expressing sNS1

Targeting NS1 for dengue vaccine development may have many advantages. The benefits of an sNS1-based dengue vaccine relate to a high degree of NS1 conservation amongst DENV serotypes (about 70% of amino-acid identity), a strong immunogenic potential of sNS1 and the evidence of an efficient anti-DENV immune response based on the stimulation of B and T cell-dependent immunity. According to the immunopathogenesis of dengue, a major advantage of using NS1 is to bypass the risk of ADE, which mainly relates to the production of antibodies that promote viral growth and severe disease. Herein, several DENV NS1-based vaccine platforms are presented and illustrated with one vaccine candidate for each platform.

The second generation of dengue vaccine candidates based on live-attenuated viruses (LAV) includes all proteins of DENV, whereas the licensed Dengvaxia elicits immunity only against the prM and E proteins as dengue antigens. The more advanced LAV dengue vaccine is tetravalent TAK-003 developed by Takeda [37]. TAK-003 consists of an attenuated DENV-2 strain together with chimeric DENV-2 in which the prM and E genes were substituted by their counterparts from DENV-1, DENV-3 and DENV-4 [37]. A single dose of the TAK-003 vaccine can elicit a durable T cell-mediated immunity against both structural and nonstructural proteins of all four DENV serotypes for at least 4 months post-immunization. Notably, TAK-003 elicits a broad response directed across the DENV-2 proteome, with focused reactivity against NS1 and NS3 [38]. The DENV-2 NS1-directed IgGs cross-react with NS1 from DENVs-1,3,4 [39]. In TAK-003 vaccines, hyperpermeability of capillary vessels and degradation of endothelial glycocalyx components were not observed regardless of the DENV serotype [39]. Consequently, the LAV vaccine provides functional NS1-specific IgG responses which confer protection against the effects of the viral toxin NS1.

**Figure 4:**
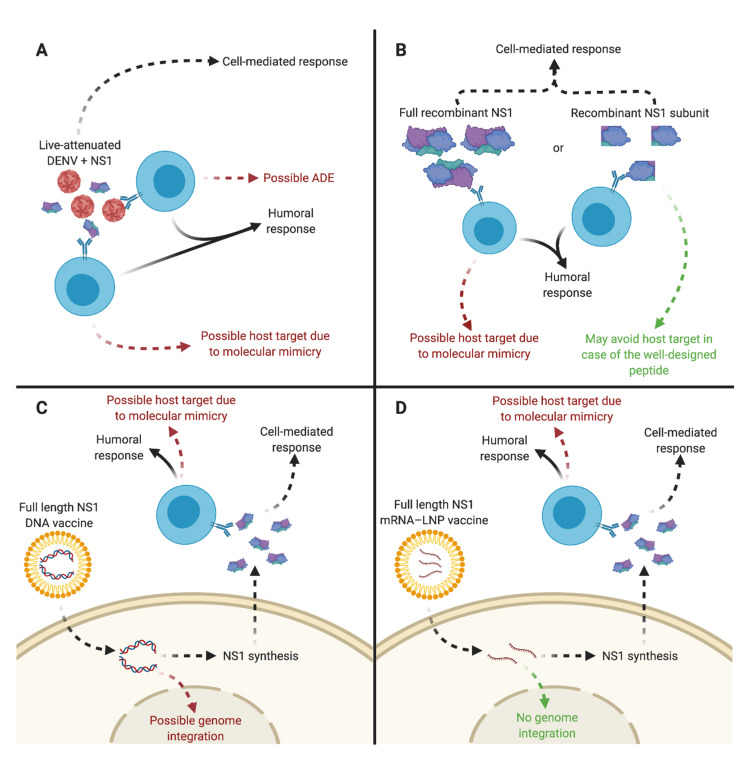
DENV NS1-based vaccine platforms. In (**A**), immunization of live-attenuated DENV (LAV) expressing NS1 induces both humoral and cell-mediated responses. However, vaccination with LAV DENV has a risk of exacerbating dengue severity via the antibody-dependent enhancement phenomenon, whereas NS1 has the ability to generate autoantibodies in relation to molecular mimicry. In (**B**), safe immunization with recombinant DENV NS1 or antigenic parts of the protein requires a lack of autoimmune antibodies. In (**C**), immunization with an encapsulated DNA vaccine expressing DENV NS1 induces both humoral and cell-mediated responses. It cannot rule out the risk of DNA integration into the host cell genome. In (**D**), encapsulated NS1 mRNA is a promising approach, with a lipid nanoparticle (LNP) playing the role of an RNA cargo transporter. Released NS1 mRNA is directly translated by the cellular machinery without the risk of integration. However, such an NS1-based vaccine should ideally avoid the risk of induction of autoantibody production.

Several technologies are currently in use to elicit an immune response against the DENV NS1 protein, as illustrated in Figure 4.

A number of DENV NS1-based vaccines have been and still are in development (Table 1).

The next generation of dengue vaccines in development, including DNA subunit, virus-like particles (VLP) and viral vector vaccines, were reviewed by Redoni et al. [52]. VLPs exhibit viral antigens with high density on their surface, providing a potential for high antigenicity and potent immunogenicity [53]. This makes VLPs a promising approach for developing safe and effective DENV vaccines. VLP-based DENV vaccines were described by Zhang et al. in 2020, but neither of them was developed with NS1 proteins [54]. One example is given with C-terminal truncated DENV-2 NS1 loaded in *N*,*N*,*N*-trimethyl chitosan nanoparticles (NS1_1–279_TMC NPs) investigated in a murine model and in humans ex vivo [41]. In a human ex vivo model, it was demonstrated that TMC particles deliver NS1_1–279_ protein in monocyte-derived dendritic cells (MODCs) and also stimulate those cells, resulting in an increased expression of maturation marker CD83, costimulatory molecules CD80, CD86 and HLA-DR and secreting diverse immune cytokines/chemokines [41]. Immunization with NS1_1–279_TMC NPs resulted in both B cell and T cell responses leading to IgG production and CD8+ T cells activation. One important finding is that DENV2 NS1_1–279_-directed antibodies have the ability to kill DENV-infected cells through antibody-dependent complement-mediated cytotoxicity [41]. Consequently, NS1-based vaccine candidates are of great interest in relation to the properties of TMC as a suitable adjuvant which enhances delivery and promotes immunogenicity of viral antigens. The NLP-associated delivery of NS1 mRNA has been assessed in preclinical studies. Injection of LNP as a carrier vehicle of mRNA expressing both the N-terminal part of E and NS1 has resulted in high levels of neutralizing DENV antibodies and viral antigen-specific T cell responses leading to complete protection against the DENV challenge.

It is well-known that interactions of the DENV NS1 protein with clotting factors and endothelial cells contribute to the immunopathogenesis of severe dengue. Several studies have identified NS1 peptide sequences that can generate anti-DENV antibodies exhibiting cross-reactivity to self-antigens [55]. Such NS1 motifs are mostly conserved across DENV serotypes and genotypes (Figure 3). For instance, it has been shown that antibodies directed against the C-terminal region of NS1 can cross-react with the LYRIC protein which localizes at the tight junctions of endothelial cells [56,57,58]. Given that immunization with a DENV NS1 peptide representing a modified LYRIC-like sequence can provide efficient protection against DENV [51], the NS1 peptides that lack mimicry sequences may represent an attractive platform in the development of NS1-based dengue vaccines.

## 5. Concluding Remarks

It is widely admitted that the threat of dengue disease requires a successful vaccine against the risk of severe dengue. An efficient dengue vaccine must be able to elicit long-term immunity to DENV regardless of the serotype or genotype of the infecting viral strain. At our stage of knowledge of the dengue disease, the key elements of the most popular dengue vaccine strategies include the notion of designing viral antigens that are targets of effective antibody-mediated neutralization of the four DENV serotypes. The Dengvaxia efficacy trials conducted to date have demonstrated that a dengue vaccine is possible and have made important contributions to our understanding of the path towards the development of such a vaccine. Since the licensed dengue vaccine Dengvaxia has shown debated efficacy, the mechanisms by which a dengue vaccine might confer efficient protection against the DENV infection need to be better defined. Consequently, new insights that will help to guide rational vaccine design against DENV are necessary. The NS1 glycoprotein has recently emerged as a potential viral antigen target for the development of dengue candidate vaccines. As a viral toxin, released soluble NS1 has been shown to play a key role in the immunopathogenesis of severe dengue. One can estimate that LAV of DENV and NS1-based vaccines represent promising strategies that have the potential to significantly advance dengue vaccine development. Given that their efficacy could be greatly improved by reducing off-target antibody responses to the DENV NS1 protein, it will be of great interest to modify using the mutational approach the NS1-associated irrelevant epitopes presenting the risk of mimicry with host factors involved in coagulation and integrity of the vascular endothelium.

## Figures and Tables

**Table 1:** Different NS1-based vaccine strategies.

Platform	Methods	Preclinical	Outcome	Reference
LAV	Tetravalent dengue vaccine (TDV)	NHP	Neutralizing anti-DENV antibodies	[40]
VLPs	NS1_1–279_TMC NPs	BALB/c mice	Protection efficacy of 97.47%	[41]
DNA	pcTPANS1 *	BALB/c mice	Protection efficacy of 100%	[42,43]
	pcENS1 **	BALB/c mice	Protection efficacy < 90%	[44]
	pE11D2 *** and pcTRANS1 ****	BALB/c mice	Protection efficacy < 90%	[45]
	pD2NS1/pD2NS1 + pIL-2	C3H mice	Protection efficacy of 50–80%	[46]
mRNA	DENV-2 E80-mRNA, NS1-mRNA	BALB/c mice	n.a.	[47]
Viral antigen	rNS1 + LTG33D adjuvant	BALB/c mice	Protection efficacy of 50%	[48]
	ΔC NS1 ^#^ + CFA adjuvant	C3H/HeN mice	Protection efficacy of 65%	[49]
	Chimeric DJ NS1 ^##^ + CFA adjuvant	C3H/HeN mice	Protection efficacy of 65%	[49]
	Full DENV1–4 NS1+ MPLA/AddaVax adjuvant	Ifnar^−/−^ C57BL6 mice	Protection efficacy of 60–100%	[9]
	Recombinant DENV NS1 + cyclic dinucleotides (CDNs) adjuvant	Ifnar^−/−^ C57BL6 mice	Protection efficacy of 60–70%	[50]
Synthetic peptide	Modified NS1-WD ^###^ + CFA adjuvant	C3H/HeN: STAT1^−/−^ C57BL6 mice	Protection efficacy of 100%	[51]

* TPA: human tissue plasminogen activator, a secretory signal sequence; ** pcENS1: encodes the C-terminal E protein and the full NS1 region; *** pE11D2: encodes the envelope (E) ectodomain (domains I, II, and III); **** pcTRANS1: encodes the nonstructural 1 (NS1) protein of DENV2; ^#^ ΔC NS1: NS1 lacking the C-terminal amino acids (a.a.) 271-352; ^##^ DJ NS1: N-terminal DENV NS1 (a.a. 1-270) and C-terminal Japanese encephalitis virus NS1 (a.a. 271-352); ^###^ NS1-WD: NS1 wing domain.

## Data Availability

Not applicable.

## Abbreviations

ADE	Antibody-dependent enhancement
DENV	Dengue virus
DHF	Dengue hemorrhagic fever
DSS	Dengue shock syndrome
E	Envelope protein
ERGIC	ER–Golgi intermediate compartment
FcγR	FC-gamma receptor
GMT	Geometric mean titer
IL	Interleukin
LAV	Live-attenuated virus
LNP	Lipid nanoparticle
LPS	Lipopolysaccharide
mAb	Monoclonal antibody
MIF	Macrophage migration inhibitory factor
MODCs	Monocyte-derived dendritic cell
NS	Nonstructural protein
PBMC	Peripheral blood mononuclear cell
prM	Precursor of the M protein
PRR	Pattern recognition receptor
TLR	Toll-like Receptor
TMC	Trimethyl chitosan
TNF-α	Tumor necrosis factor α
VLP	Virus-like particle
WD	Wing domain
WNV	West Nile virus
ZIKV	Zika virus
